# Development of A Novel Scoring Index to Quantify the Need for Nutritional Support Interventions During Stem Cell Transplant and Gene Therapy in Children

**DOI:** 10.1111/jhn.70124

**Published:** 2025-09-14

**Authors:** Gerard Gurumurthy, Rebecca Dixon, Victoria Whiteley, Claire Horgan, Omima Mustafa, Nicola Williams, Robert Wynn

**Affiliations:** ^1^ The University of Manchester Manchester UK; ^2^ Royal Manchester Children's Hospital Manchester UK

**Keywords:** children, chimeric antigen receptor T‐cell therapy, gene therapy, haematopoietic stem cell transplant, nutrition assessment, parenteral nutrition

## Abstract

**Background:**

Paediatric patients undergoing stem cell and gene therapy frequently experience nutritional challenges. Although nutritional status is known to correlate with outcomes, there is no scoring index to quantify the intensity and duration of dietetic interventions in children. We developed a nutritional intensity score that assigns weighted values to different nutritional support strategies that reflects both intervention type and duration over the first 100 days post‐transplant.

**Methods:**

We retrospectively analysed 131 paediatric patients after transplant or gene therapy. Nutritional interventions were categorised into five levels: routine dietetic review (1 point), oral nutritional support (2 points), enteral nutritional support (4 points), parenteral nutritional (PN) support (6 points), and bespoke PN support (7 points). The cumulative nutritional intensity score was calculated by multiplying the intervention points by the duration (in days) and summing across all interventions. Subgroup analyses compared median area under the curve (AUC) values in different groups. A further multivariate logistic regression was employed to assess predictors of ≥ PN need at Day 100 within the allogeneic group.

**Results:**

Nutritional intensity varied with different treatment modalities. Chimeric Antigen Receptor T‐cell (CAR‐T) patients showed notably lower median nutritional intensity score (53.0, 95% CI: 30–117) relative to allogeneic (326.0, 95% CI: 282–404) or stem cell gene therapy (124.5, 95% CI: 56–182, *p* < 0.05) recipients. Within the allogeneic group (*n* = 109), patients with malignant diseases had a median nutritional intensity score of 444.0 (95% CI: 350.0–466.0) compared to 261.5 (95% CI: 224.0, 322.0, *p* < 0.01) in those with nonmalignant conditions. Those receiving total body irradiation (TBI) had higher median nutritional intensity score values (476.0, 95% CI: 317–543) than those without TBI (302.0, 95% CI: 244–386; *p* < 0.01). In the multivariate analysis, higher nutritional intensity score, malignant disease status, and TBI were significant predictors of ≥ PN requirement at Day 100.

**Conclusion:**

Our nutritional intensity score reflects the cumulative burden of nutritional interventions in paediatric patients, and will inform patient and service planning. Future prospective studies are required to validate its predictive value.

## Summary

- An area under the curve (AUC)‐derived nutritional intensity score was developed to integrate both the intensity and duration of dietetic interventions over the first 100 days post‐transplant. It assigned weighted values for routine review, oral, enteral, parenteral, and bespoke parenteral support.
- In children undergoing allogeneic stem cell transplant, those with malignant disease or conditioned with total body irradiation exhibited significantly higher nutritional intensity scores.
- A higher nutritional score independently predicted the need for prolonged parenteral nutrition at Day +100 (OR = 2.43 per 100‐unit increase). This may inform early risk stratification and resource allocation for those needing prolonged PN.

## 1 Introduction

Paediatric patients undergoing hematopoietic stem cell transplantation (HSCT) or cellular therapies, such as chimeric antigen receptor T‐cell (CAR‐T) therapy and autologous gene therapy, experience nutritional challenges [1]. Conditioning regimens involving high‐dose chemotherapy, with or without total body irradiation (TBI), and the occurrence of post‐transplant complications (e.g., mucositis, graft‐vs.‐host disease, and infections) contribute to significant reductions in oral intake [2, 3, 4, 5, 6]. The ensuing nutritional failure is a common comorbidity in patients undergoing HSCT and is associated with adverse clinical outcomes [7, 8]. These include prolonged hospitalisation, increased infection risk, and higher transplant‐related mortality [9, 10, 11].

Adequate nutritional support is critical during the prehabilitation and peri‐transplant period as it can enhance immune reconstitution, mitigate infection risks, and improve overall recovery [10, 12]. The spectrum of nutritional interventions ranges from routine dietetic reviews to advanced measures such as parenteral nutrition (PN) and bespoke PN solutions [13]. Recent clinical guidelines have increasingly emphasised the preferential use of enteral nutrition over PN whenever possible, given its role in maintaining gut integrity and reducing complications [14, 15, 16].

Despite the recognised importance of nutritional support in paediatric HSCT and cellular therapies, the absence of a standardised method to quantify dietetic intervention intensity remains a significant gap. Most studies report nutritional support without capturing the nuanced intensity or cumulative burden of nutritional care. One such score, using a weekly nutrition summary score, had demonstrated a correlation with outcomes but lacked a time‐weighted, integrative approach [17].

To address this, we developed an Area Under the Curve (AUC)‐based dietetic intensity score that assigns weighted values to each level of nutritional intervention and multiplies them by the duration (in days) of the intervention over the first 100 days post‐transplant. The nutrition intensity score was designed to [1] stratify patients according to their risk of requiring prolonged, high‐intensity nutritional support, and [2] inform data‐driven allocation of dietetic and clinical resources. By weighting both the level and duration of each nutritional modality, the score captures patient‐experienced burden (the cumulative duration and invasiveness of support) alongside service‐level burden, including total dietetic full‐time equivalent days and consumable costs.

In this report, we aim to describe the development of this scoring index, compare the median cumulative intensities across various subgroups, and explore the association between higher nutritional support intensity and the need for PN at Day 100.

## 2 Methods

### 2.1 Study Design and Patient Selection

We conducted a retrospective, single‐centre study including paediatric patients (age < 18 years) who underwent HSCT, CAR‐T, or stem cell gene therapy between September 2020 and January 2025. Patients were included if complete dietetic records were available for the 100‐day post‐transplant period. Individuals lost to follow‐up or died < 100 days were excluded from the analysis. All patients are consented with institutional consent to anonymised data use in service and outcome evaluations.

### 2.2 Data Extraction

Clinical data, including demographic information, disease type (malignant vs. nonmalignant), transplant modality (allogeneic, CAR‐T, gene therapy), cell source (cord blood vs. others), and use of total body irradiation (TBI), were extracted from the electronic health record. Dietetic interventions were categorised into five groups: routine dietetic review (monitoring without active physical nutritional intervention), oral nutritional support, enteral nutritional support, parenteral nutritional support, and bespoke parenteral nutritional support.

### 2.3 Derivation of the Nutritional Support Intensity Model

**Figure 1:**
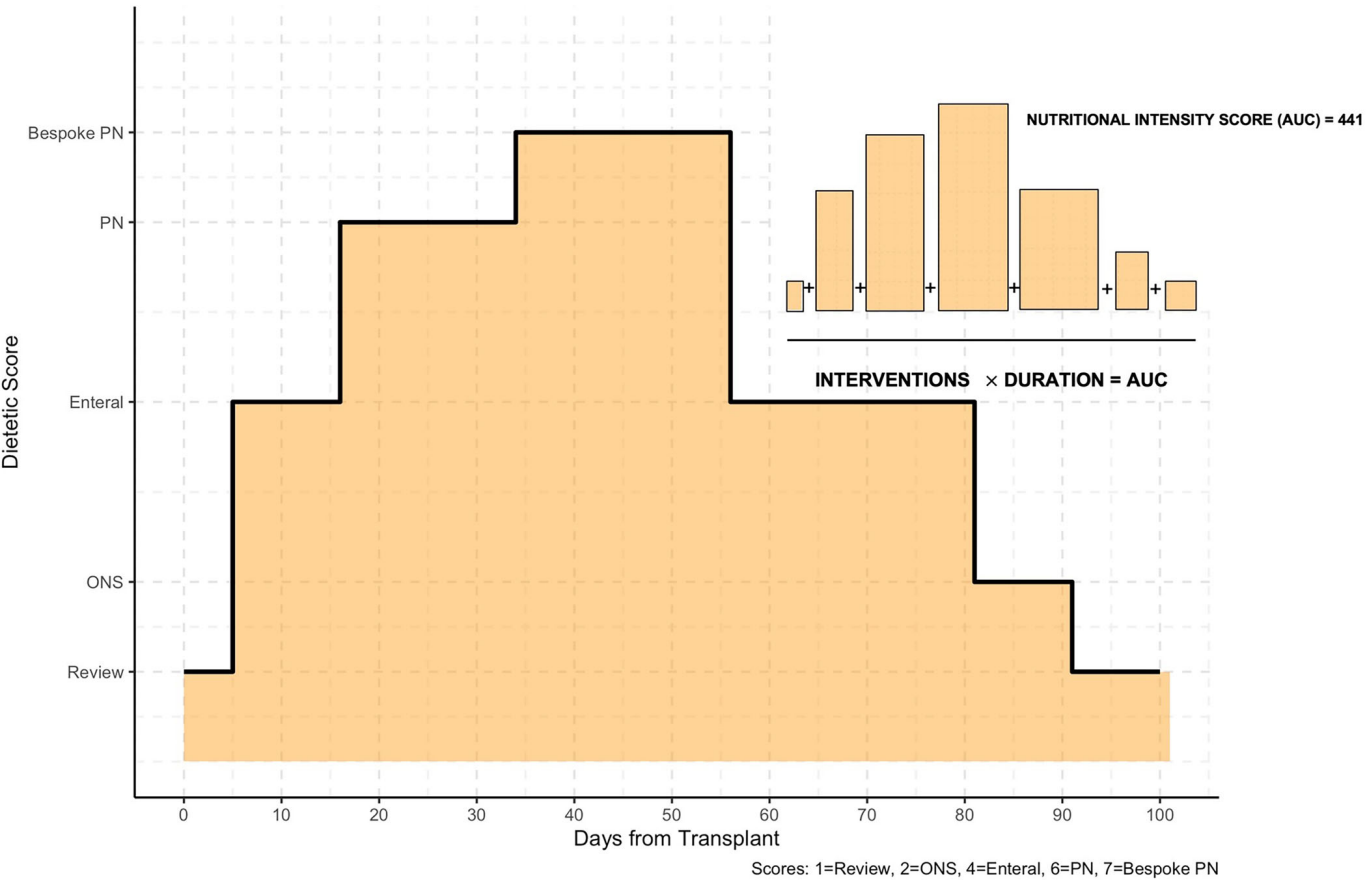
Derivation of nutritional intensity score (Area Under the Cure, AUC).

Each type of dietetic intervention was assigned a predefined intensity score as follows: routine dietetic review (1 point), oral nutritional support (2 points), enteral nutritional support (4 points), parenteral nutritional support (6 points), and bespoke parenteral nutritional support (7 points). For each patient, the intensity score associated with each intervention was multiplied by the number of days that particular support was administered, represented as:

$${\mathrm{AUC}}_{i}=\sum_{j=1}^{n}\left(\mathrm{Intensity}\;{\mathrm{Score}}_{\mathrm{ij}}+\mathrm{Duration}\;\mathrm{of}\;\mathrm{Dietetic}\;{\mathrm{Intervention}}_{\mathrm{ij}}\right)$$

where ${\text{AUC}}_{i}$ is the cumulative area under the curve for patient i, denotes the intensity score for intervention j given to the patient, and ${\text{Duration}}_{\mathrm{ij}}$ is the number of days the intervention was applied. The final nutritional intensity score for each patient was the sum of these products across all intervention types during the 100‐day period (Figure 1).

We selected an AUC‐based approach to derive our cumulative intensity score as it integrates both the level and duration of support into a single continuous metric, avoids arbitrary early vs. late cut‐offs or dichotomisation, and remains robust to variable lengths of stay. The weights assigned to each level of support were chosen to mirror the escalating clinical effort from minimal oversight with routine reviews through pharmacy compounding and sterile‐room requirements for bespoke PN. The end result is a score that quantitatively captures both the level and duration of nutritional support.

### 2.4 Endpoints

The primary endpoint of this study was defined as the intensity of nutritional intervention, expressed as the cumulative AUC, at 100 days post‐transplant, with a particular focus on whether patients remained under active dietetic review and the level of support being administered. The Day +100 cut‐off was chosen as it represents the standard timepoint for reporting early transplant‐related morbidity.

Subgroup analyses stratify patients by age (< 2 years, 2‐12 years, and > 12 years), disease type (malignant vs. nonmalignant), conditioning (with vs. without total body irradiation) and transplant modality (allogeneic vs. CAR‐T vs. autologous gene therapy).

### 2.5 Statistical Analysis

Median cumulative nutritional intensity score and 95% confidence intervals were determined for subgroups defined by gender (male vs. female), age (< 2 years vs. 2–12 years vs. > 12 years), disease type (malignant vs. nonmalignant), transplant type (allogeneic vs. CAR‐T vs. gene therapy), cell source (cord blood vs. others), and conditioning (TBI used vs. not used). Intergroup comparisons were made using analysis of variance (ANOVA) for groups of three or more and independent *t*‐tests for dichotomous comparisons. In addition, the association between nutritional intensity (AUC) and the requirement for ≥ PN at Day 100 was evaluated using both bivariable and multivariable logistic regression models. Statistical analyses were conducted using R (version 4.4.3, R Foundation for Statistical Computing, Vienna, Austria. Available at https://www.R-project.org). For all tests, the significance level was set at *p* < 0.05.

## 3 Results

### 3.1 Baseline Characteristics

A total of 131 paediatric patients were included in the study (Table 1). The median age was 7.0 years (95% CI: 4.0–8.0), and 65.6% (*n* = 86) of the patients were male. Patients underwent various transplant modalities: allogeneic (*n* = 109, 83.2%), CAR‐T (*n* = 16, 12.2%), and gene therapy (*n* = 6, 4.6%).

**Table 1:** Baseline demographic data (*n* = 131).

Demographic	*N* = 131
Age	
Median Age (IQR)	7 (3–12)
< 2	27 (20.6%)
2–12	76 (58.0%)
> 12	28 (21.4%)
Gender	
Male	86 (65.6%)
Female	45 (34.4%)
Transplant type	
Allogeneic	109 (83.2%)
CAR‐T	16 (12.2%)
Stem Cell Gene therapy	6 (4.6%)
*Of those undergoing allogeneic transplant*	*N* = 109
Allogeneic cell source	
Cord Blood	31 (28.4%)
Others	78 (72.6%)
Disease	
Malignant	45 (41.3%)
Acute lymphoblastic leukaemia	20 (44.4%)
Acute myeloid leukaemia	16 (35.6%)
Juvenile myelomonocytic leukemia	5 (11.1%)
Myelodysplastic syndromes	4 (8.9%)
Nonmalignant	64 (59.7%)
Conditioning	
Total body irradiation	14 (12.8%)
Not irradiated	95 (87.2%)

### 3.2 Nutritional Intensity

**Figure 2:**
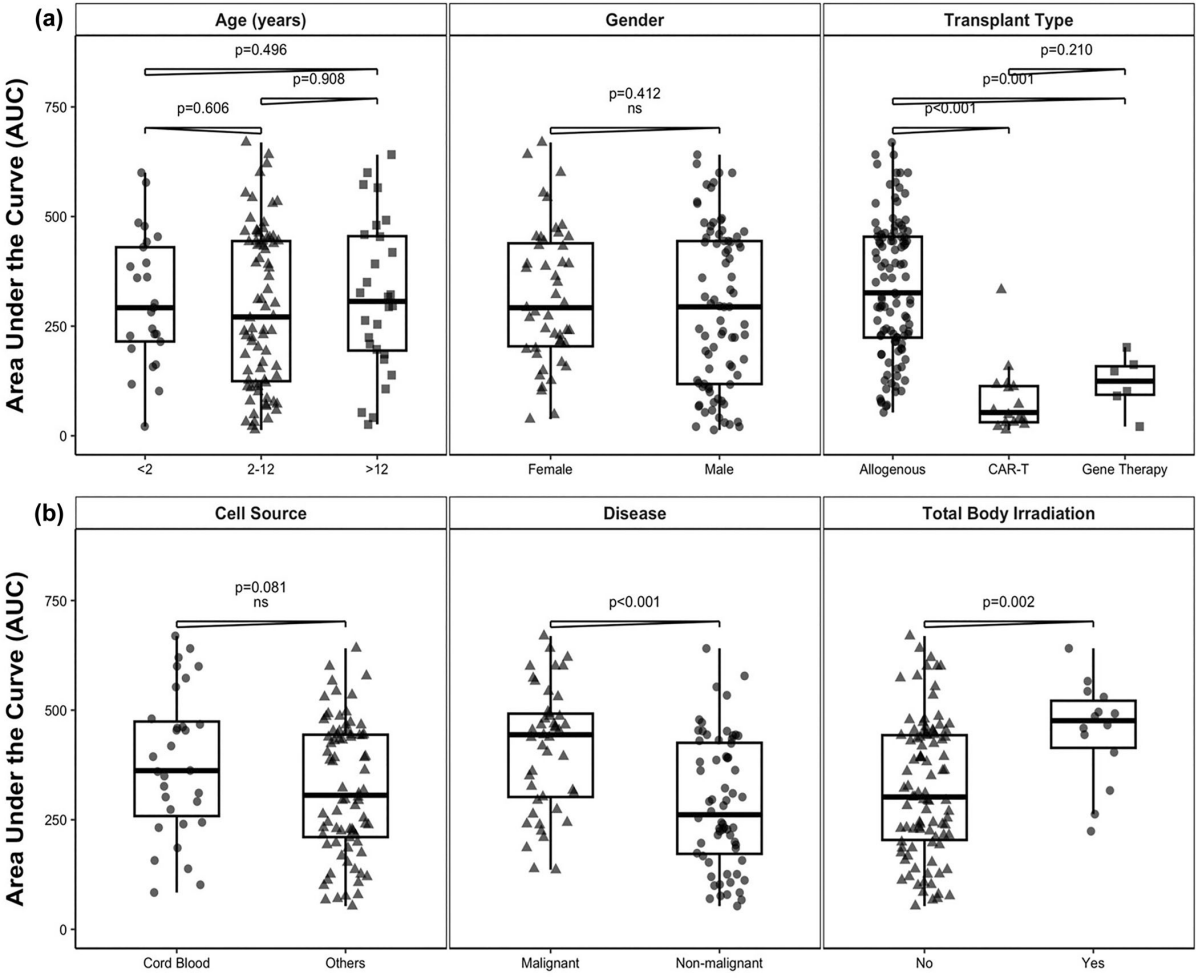
Analysis of Median Nutritional Intensity Score in Subgroups. (a) Area Under the Curve (AUC) of dietetic interventions across various patient demographics and treatment characteristics, including age, gender, and transplant type. (b) Within allogeneic group (*n* = 109), significant differences in median AUC were observed, with malignant disease (*p* < 0.001) and TBI use (*p* = 0.002) were associated with higher dietetic intervention intensity.

The median nutritional intensity score (AUC) over 100 days for the entire cohort was 294.0 (95% CI: 232.0–360.0). The cumulative nutritional intensity score was compared across subgroups (Figure 2). There were no significant differences in median nutritional intensity between males versus females (294.0, 95% CI: 224.0–362.0 vs. 292.0, 95% CI: 232–391, *p* = 0.418) and age groups < 2, 2–12, and > 12 years (292.0, 95% CI: 228.0–394.0 vs. 271.0, 95% CI: 193.0–394.0 vs. 306.5, 95% CI: 209.0–418.0 respectively; *p* = 0.496). Comparisons based on transplant modality showed that allogeneic transplant recipients had a significantly higher median cumulative nutritional intensity score (326.0, 95% CI: 282.0–404.0) compared to CAR‐T (53.0, 95% CI: 30.0–117.0) and gene therapy groups (124.5, 95% CI: 56–182, *p* < 0.05).

Of the 109 allogeneic patients, those receiving cord blood had a higher median cumulative nutritional intensity score compared to those with other sources, although this was not significant (362.0, 95% CI: 292.0–462.0 vs. 306.0, 95% CI: 240.0–404.0; *p* = 0.081). Within the allogeneic cohort, eight patients developed gut GvHD, giving an incidence of 7.8% (95%CI 3.4–14.7). Allogeneic patients with malignant diseases had a significantly higher median cumulative nutritional intensity score compared to patients with nonmalignant conditions (444.0, 95% CI: 350.0–466.0 vs. 261.5, 95% CI: 224.0–322.0; *p* < 0.01). Patients who received TBI for conditioning demonstrated a significantly higher median cumulative nutritional intensity score in contrast to those who did not (476.0, 95% CI: 317.0–543.0 vs. 302.0, 95% CI: 244.0–386.0; *p* < 0.01).

### 3.3 Subgroup analysis: Allogeneic Patients at High‐risk of Requiring ≥ Parenteral Support at Day + 100

In subset analysis of 109 allogeneic transplant patients, 62.4% (*n* = 68) required ≥ PN at Day +100. Exploratory analysis determined differences in ≥ PN need across predictors. Disease type showed a significant difference, with 86.7% of patients with malignancies requiring ≥ PN compared to 45.3% of patients with non‐malignancies (*p* < 0.001). Patients with cord blood as the cell source had a higher ≥ PN need (80.6%) than those with other sources (55.1%, *p* < 0.05). The median cumulative nutritional intensity score was significantly higher in patients requiring ≥ PN than in those who did not (433.0 vs. 215.0, *p* < 0.001). There were no significant differences in the proportion of ≥ PN needed at 100 days between gender, age groups, and TBI conditioning (*p* > 0.05).

**Figure 3:**
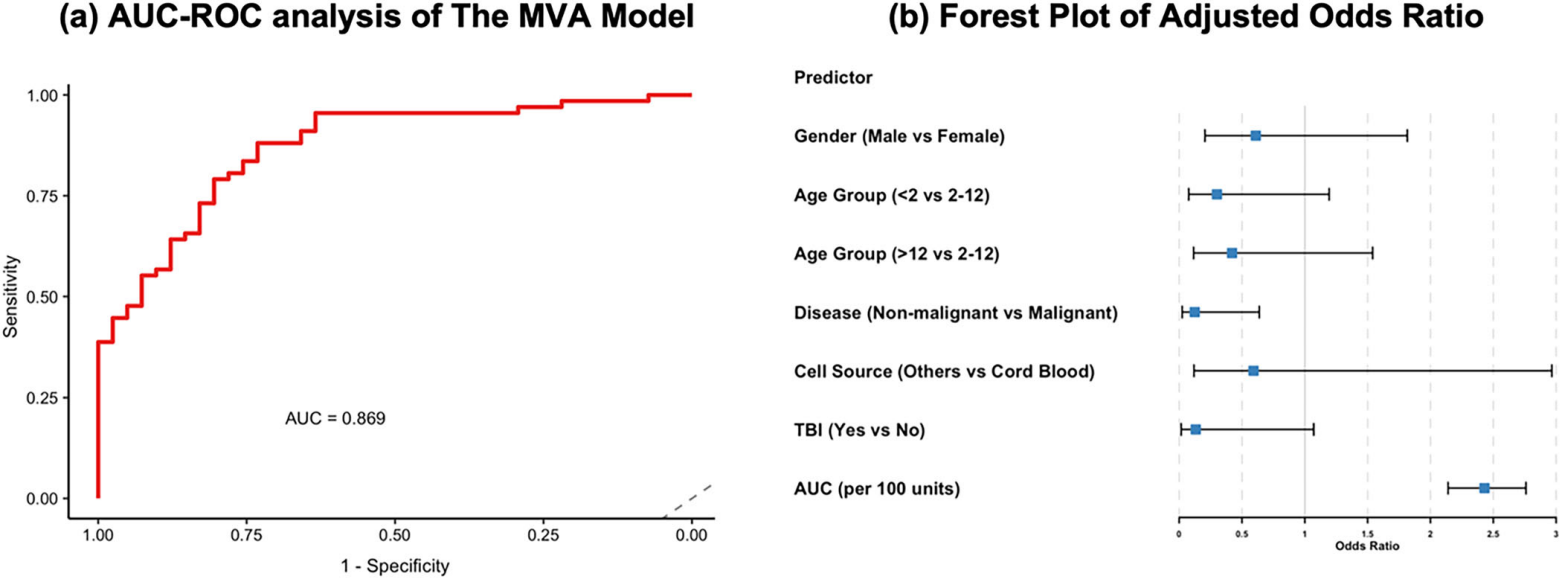
Multivariable Analysis (MVA) of Parenteral (PN) Need (both standard and bespoke) at Day 100 Post‐Transplant. (a) Model Discrimination. The receiver operating characteristic (ROC) curve for the multivariable logistic regression model demonstrates high discrimination, with an area under the curve (AUC‐ROC) of 0.869. (b) Forest Plot of Adjusted Odds Ratios. The analysis determined that malignant disease (OR: 0.124, 95% CI: 0.024–0.638, *p* < 0.05) and higher nutritional intensity (OR: 1.009 per unit, 95% CI: 1.005–1.013, *p* < 0.001) are significant independent predictors of PN need. TBI showed a trend toward significance (OR: 0.132, 95% CI: 0.016–1.071, *p* = 0.054), while gender, age group, and cell source did not reach statistical significance.

A multivariable logistic regression model (Table 2 and Figure 3) incorporating gender, age group, disease type, cell source, TBI, and cumulative nutritional intensity score further confirmed that malignant disease (OR: 0.124, 95% CI: 0.024–0.638, *p* < 0.05) and higher nutritional intensity remained independent predictors of PN need. The odds increased by approximately 2.43‐fold for every 100 unit increase in AUC (OR: 1.009 per unit, 95% CI: 1.005–1.013, *p* < 0.001). TBI showed a trend towards significance (OR; 0.132, 95% CI: 0.016–1.071, *p* = 0.054). Age and cell source did not reach statistical significance in the multivariable model. The model achieved a high discriminatory performance (AUC‐ROC = 0.869).

**Table 2:** Multivariable Logistic Regression Results. Malignancies and higher nutritional intensity score (AUC) are significant predictors of needing parenteral or bespoke parenteral support at 100 days post‐transplant. Total body irradiation showed a trend towards significance.

Predictor	Estimate	Odds ratio	95% CI	*p*‐value
Intercept	0.561	1.753	0.217–14.149	0.590
Gender (male vs. female)	−0.494	0.610	0.205–1.816	0.375
Age group (< 2 vs. 2–12)	−1.200	0.301	0.076–1.194	0.088
Age group (> 12 vs. 2–12)	−0.866	0.421	0.115–1.540	0.191
Disease (nonmalignant vs. malignant)	−2.091	0.124	0.024–0.638	0.013
Cell source (others vs. cord blood)	−0.524	0.592	0.118–2.966	0.524
TBI (yes vs. no)	−2.028	0.132	0.016–1.071	0.054
AUC (per unit)	0.009	1.009	1.005–1.013	< 0.001

## 4 Discussion

Our study introduces an AUC‐derived nutritional intensity score designed to quantify the cumulative nutritional support burden in paediatric patients undergoing allogeneic transplant, CAR‐T, or gene therapy. Our analysis revealed that allogeneic patients with malignant diseases or those conditioned with TBI exhibited significantly higher nutritional intensity scores. In contrast, patients undergoing CAR‐T or autologous gene therapy had markedly lower scores. This finding aligns with differences in conditioning intensity, engraftment kinetics, and post‐transplant complications [18, 19, 20].

The association between TBI and higher nutritional support needs may be mediated by the mucosal injury and systemic inflammatory response that TBI elicits. TBI, as part of myeloablative conditioning, can cause extensive oral and gastrointestinal mucositis [3, 21]. The resultant pain, dysphagia, and diarrhoea may severely impair oral intake. This would necessitate prolonged reliance on parenteral nutrition [6, 22, 23].

Cord blood as a cell source exhibited a trend towards significance with higher nutritional burden. Ideally, a comparison across all the different cell sources would more precisely delineate differences in nutritional support requirements. However, our limited sample size in each cell source group hindered this approach. Instead, separating cord blood vs. all other sources offers a pragmatic alternative. Umbilical cord blood transplants typically exhibit slower engraftment compared to bone marrow or peripheral blood transplants, resulting in prolonged neutropenia and extended hospitalisation [24]. The longer duration during which patients remain dependent on artificial nutritional support explains the higher cumulative nutritional intensity scores observed in this subgroup.

Moreover, our comparison of transplant modalities demonstrated that allogeneic HSCT patients have significantly higher nutritional support requirements than those receiving CAR‐T or gene therapy. Allogeneic transplants require intensive myeloablative regimens that cause severe gastrointestinal toxicity and predispose patients to complications such as graft‐versus‐host disease (GvHD). GvHD, particularly when involving the gastrointestinal tract, results in malabsorption and protracted feeding difficulties, leading to sustained nutritional support needs [25, 26]. In contrast, CAR‐T and autologous gene therapy employ less intensive conditioning protocols, thereby permitting a more rapid recovery of oral intake and lower overall nutritional support intensity.

Our multivariate logistic regression analyses further corroborate that higher cumulative nutritional support, as captured by our cumulative nutritional intensity score, is a strong independent predictor of the need for prolonged parenteral nutrition in the allogeneic population. This relationship suggests that the dietetic intensity score not only reflects current practice but may also serve as a prognostic marker for nutritional complications. The strong association between high cumulative nutritional intensity scores and adverse nutritional outcomes, particularly in the context of malignant disease and intensive conditioning regimens, underscores the clinical relevance of our scoring system.

Clinically, this score can serve as an objective metric for early identification of paediatric patients who are at risk of nutritional compromise and prolonged need for artificial nutrition. Our data suggest that paediatric allogeneic patients who undergo TBI conditioning, cord blood transplant, or who are being treated for malignant disorders as high‐risk for requiring more intense nutritional support, potentially up to +100 days post‐transplant. By quantifying the cumulative burden of nutritional interventions, multidisciplinary teams can stratify patients based on their risk profiles and allocate resources more effectively. For instance, a high AUC score early in the post‐transplant period may prompt earlier and more aggressive nutrition support. It could also help with setting parental expectations and future acceptance of more intensive interventions earlier. This would allow for a timely initiation of enteral or parenteral nutrition, tailored to individual patient needs. Additionally, this index could be incorporated into clinical decision‐making algorithms that guide the escalation or de‐escalation of nutritional therapies. Monitoring changes in the score over time may also serve as a quality improvement measure, enabling clinicians to benchmark nutritional care and adjust protocols to minimise complications. By standardising the assessment of nutritional support, this tool may facilitate more personalised and dynamic management strategies in paediatric transplant settings.

Several limitations require consideration. While the AUC‐derived cumulative nutritional intensity score offers a time‐weighted metric, it may simplify the dynamic nature of nutritional support into discrete point assignments and day counts. Although we tried to align each weight with relative resource use and clinical complexity, these assignments may be viewed as subjective and institution‐specific. Additionally, different centres may employ alternative feeding protocols or staffing models which potentially limits direct comparability. The small sample sizes for the CAR‐T and gene therapy cohorts limit their comparison of nutritional intensity against the larger allogeneic cohort. We were also unable to undertake subgroup analyses for these two cohort due to the sample size. While our study is limited by its retrospective and single‐centre design, the generalisability of the findings may aid clinicians in identifying and providing more individualised dietetic intervention for those in need. Prospective validation will be essential for assessing whether this index can improve clinical outcomes by facilitating early, targeted nutritional interventions.

## 5 Conclusion

We introduce an AUC‐based dietetic intensity scoring index that quantitatively captures the cumulative burden of nutritional support in paediatric patients undergoing allogeneic transplant, CAR‐T, and autologous gene therapy. Our findings suggest that higher nutritional intensity is associated with malignant disease, TBI conditioning, and cord blood grafts. Lower scores are seen in patients receiving CAR‐T or gene therapy. The score's strong correlation with prolonged parenteral nutrition underscores its potential utility as a clinical tool for early risk stratification and intervention for those needing prolonged PN. Ultimately, the implementation of this scoring system may enable more tailored, proactive nutritional management. Future, prospective studies will be crucial to validate the utility of the score and to further integrate nutritional monitoring into paediatric transplant care.

## Author Contributions

**Gerard Gurumurthy:** data collection, data analysis, writing – original draft. **Rebecca Dixon:** data collection, data analysis, writing – review and editing. **Victoria Whiteley:** writing – review and editing. **Claire Horgan:** writing – review and editing. **Omima Mustafa:** writing – review and editing. **Nicola Williams:** data analysis, writing – review and editing **Robert Wynn:** data analysis, writing – review and editing, conceptualisation.

## Ethics Statement

All patients are consented with institutional consent to anonymised data use in service and outcome evaluations.

## Consent

The authors have nothing to report.

## Conflicts of Interest

The authors declare no conflicts of interest.

## Data Availability

Data supporting the findings of this study are available within the paper.
